# A hinge position distal to the adductor tubercle minimizes the risk of hinge fractures in lateral open wedge distal femoral osteotomy

**DOI:** 10.1007/s00167-020-06244-6

**Published:** 2020-08-24

**Authors:** Philipp W. Winkler, Marco C. Rupp, Patricia M. Lutz, Stephanie Geyer, Philipp Forkel, Andreas B. Imhoff, Matthias J. Feucht

**Affiliations:** 1 Department for Orthopedic Sports Medicine, Klinikum Rechts Der Isar, Technical University of Munich, Ismaninger Str. 22, 81675 Munich, Germany; 2 Department of Orthopedics and Trauma Surgery, Medical Center, Faculty of Medicine, Albert-Ludwigs-University of Freiburg, Freiburg, Germany

**Keywords:** Open wedge, Distal femoral osteotomy, Hinge fracture, Safe zone, Target point, Valgus deformity

## Abstract

**Purpose:**

To evaluate the incidence and morphology of medial cortical hinge fractures in lateral open wedge distal femoral osteotomy (LOW-DFO) and to determine a safe zone for the position of the osteotomy hinge to minimize the risk of hinge fractures.

**Methods:**

Consecutive patients who underwent LOW-DFO for symptomatic valgus malalignment were screened for eligibility for this retrospective observational cohort study. Demographical and surgical data were collected. The incidence and morphology of medial cortical hinge fractures were evaluated on standard postoperative anterior–posterior knee radiographs. Comprehensive measurements evaluating the osteotomy gap and the position of the osteotomy hinge were taken. Additionally, each osteotomy hinge was assigned to a corresponding sector of a proposed five-sector grid of the distal medial femur.

**Results:**

A total of 100 patients (60% female) with a mean age of 31 ± 13 years were included. The overall incidence of medial cortical hinge fractures was 46% and three distinct fracture types were identified. The most frequently observed fracture type was extension of the osteotomy gap (76%), followed by a proximal (20%) and distal (4%) course of the fracture line in relation to the hinge. Group comparison (hinge fracture vs. no hinge fracture) showed statistically significant higher values for the height of the osteotomy gap (*p* = 0.001), the wedge angle (*p* = 0.036), and the vertical distance between the hinge and the proximal margin of the adductor tubercle (AT; *p* = 0.002) in the hinge fracture group. Furthermore, a significantly lower horizontal distance between the hinge and the medial cortical bone (*p* = 0.036) was observed in the hinge fracture group. A statistically significant higher incidence of medial cortical hinge fractures was observed when the position of the osteotomy hinge was proximal compared to distal to the proximal margin of the AT (53% vs. 27%; *p* = 0.023).

**Conclusion:**

Medial cortical hinge fractures in LOW-DFO are a common finding with three distinct fracture types. To minimize the risk of medial cortical hinge fractures, it is recommended to aim for a position of the osteotomy hinge at the level of or distal to the proximal margin of the adductor tubercle.

**Level of evidence:**

Prognostic study; Level III

## Introduction

The correction of valgus malalignment is indicated for the treatment of lateral compartment osteoarthritis (OA) [2, 3, 6, 30, 34], patellofemoral disorders [5, 11, 27, 32], and in combination with cartilage regenerative or meniscus replacing procedures of the lateral compartment [2]. Varus producing osteotomies are usually performed at the distal femur in a medial closed wedge (MCW) or the lateral open wedge (LOW) technique [9, 36].

Good functional, clinical, and radiographic outcomes as well as a reported survivorship of almost 90% at 5 years and more than 70% at 10 years have made the LOW distal femoral osteotomy (DFO) an attractive technique for the treatment of symptomatic valgus deformity [2, 3, 6, 7, 17, 34]. Encouraging clinical outcomes are supported by a recently published biomechanical study, which has demonstrated the unloading effect of the lateral compartment after LOW-DFO [37]. However, some disadvantages, which are inherently related to the LOW technique, are reported and discredit this technique. The most commonly reported drawbacks include a high rate of reoperations and a considerable number of delayed and non-unions of the osteotomy gap [2, 3, 6, 14, 20, 30]. One reason for non-unions could be a fracture of the medial cortical hinge and the associated reduced axial and torsional stiffness as well as the increased rotational movement across the osteotomy gap for the bone-implant construct [1, 29]. Therefore, fractures of the medial cortical hinge should be avoided. Safe zones for the position of the osteotomy hinge have been described for medial open wedge high tibial osteotomies (HTO) [13, 24, 28] and MCW-DFOs [15, 26] to minimize the risk of cortical hinge fractures. However, analyses of medial cortical hinge fractures in LOW-DFO and the definition of a safe zone for the osteotomy hinge are missing in the current literature.

Therefore, the primary objective of this retrospective observational cohort study was to evaluate the incidence and morphology of medial cortical hinge fractures in LOW-DFO. A further objective was to determine a safe zone for the position of the osteotomy hinge to minimize the risk of medial cortical hinge fractures. It was hypothesized that medial cortical hinge fractures in LOW-DFO are a common finding and that the risk of medial cortical hinge fractures increases with a more proximal position of the osteotomy hinge.

## Methods

Radiographs of 127 consecutive patients who underwent LOW-DFO for symptomatic valgus malalignment between 2015 and 2019 were screened for eligibility for this retrospective observational cohort study. Closed physes, medical records, and postoperative standard anterior to posterior (AP) and lateral knee radiographs were required for inclusion. Patients who had a history of previous osteotomies, fractures, or posttraumatic deformities of the distal femur were excluded from this study. A concomitant correction of a torsional deformity of the distal femur, which is associated with an iatrogenic disruption of the medial cortical bone, led to exclusion of the study. Additionally, patients with a severely malrotated postoperative AP knee radiograph, which was accompanied by a misprojection of the bony landmarks, especially the adductor tubercle (AT) [21], were excluded. Twenty-seven patients did not meet the inclusion criteria (1 posttraumatic deformity, 6 concomitant torsional osteotomies, 20 malrotated radiographs without visualization of the AT). Thus, a total of 100 patients could be included in the present study. A LOW-DFO was performed due to lateral compartment OA or cartilage defects in 50 (50%) patients, patellofemoral maltracking associated with patellofemoral instability or OA in 37 (37%) patients, and chronic ligamentous insufficiency in 13 (13%) patients. A review of the medical records was conducted to collect demographical and surgical data. A comprehensive summary of the descriptive statistics of the demographical and surgical data is presented in Table 1.

**Table 1:** Descriptive statistics of the demographical and surgical data of the total study group

Variable	Total study group
Number of included patients, *n*	100
Age^a^ (years)	31.3 ± 12.7 (15–64)
BMI (kg/m^2^)	26.4 ± 4.9 (16.1–35.9)
Sex	
Male, *n* (%)	40 (40%)
Female, *n* (%)	60 (60%)
Laterality	
Right, *n* (%)	57 (57%)
Left, *n* (%)	43 (43%)
Hinge fracture	
Yes, *n* (%)	46 (46%)
No, *n* (%)	54 (54%)
Fracture morphology^b^	
Type 1 (extension), *n* (%)	35 (76.1%)
Type 2 (distal), *n* (%)	2 (4.3%)
Type 3 (proximal), *n* (%)	9 (19.6%)
Concomitant procedures^c^	
None, *n* (%)	48 (48%)
HTO-MCW, *n* (%)	4 (4%)
Ligament surgery, *n* (%)	36 (36%)
PF prosthesis, *n* (%)	7 (7%)
Cartilage surgery, *n* (%)	10 (10%)
Trochleaplasty, *n* (%)	2 (2%)
Complications	
None, *n* (%)	95 (95%)
Infection, *n* (%)	2 (2%)
Non-union, *n* (%)	2 (2%)
Traumatic femur fracture, *n* (%)	1 (1%)

Categorical variables are presented as count and percentage; Continuous variables are presented as mean ± standard deviation (range)

*BMI*, body-mass-index; *HTO-MCW*, high tibial osteotomy medial closed wedge; *PF*, patellofemoral

^a^Age at surgery

^b^Hinge fracture group (*n* = 46)

^c^Total number of patients exceeds 100 (total study group), 7 patients had two concomitant procedures

Data collection and analysis was performed between December 2019 and April 2020. The approval to conduct this study was granted by the ethical review committee of the Technical University of Munich (No. 6/20S).

### Indications and surgical technique

A comprehensive preoperative analysis of the valgus malalignment based on AP hip-knee-ankle radiographs was followed by a detailed planning of the osteotomy using the medical software mediCAD® (mediCAD Hectec GmbH, Altdorf, Deutschland). A postoperative mechanical leg axis crossing the center of the tibial plateau (50% from medial to lateral) was the desired amount of correction. A step-by-step manual of the performed surgical procedure was previously described in detail [9]. A locking compression plate, PEEKPower™ Plate (Arthrex Inc., Naples, FL, USA) or TomoFix™ Plate (DePuy Synthes, Raynham, MA, USA), was used to secure the osteotomy gap. No bone grafting of the osteotomy gap was performed among the included patients. The rehabilitation program started at the first postoperative day and was dependent on the primary diagnosis and the concomitant procedures.

### Medial cortical hinge fracture

A hinge fracture was defined as a disruption of the medial cortical bone. Two observers (P.W.W., M.J.F.) independently assessed each postoperative AP knee radiograph for the presence of a medial cortical hinge fracture. In cases of disagreement, a third observer (M.C.R.) was consulted to achieve consensus. Additionally, the fracture morphology of all medial cortical hinge fractures was evaluated and a classification was established. Each hinge fracture was assigned to the respective fracture type in order to determine the incidence of each type.

### Postoperative measurements and hinge position

For postoperative measurements, standard AP knee radiographs were used, which were acquired on the first or second postoperative day. All measurements were obtained by the main observer (P.W.W.) using the picture archiving and communication system (PACS). For twenty randomly selected patients, measurements were taken twice at an interval of one month by the main observer (P.W.W.) and additionally once by a second observer (M.C.R.) to determine the inter- and intrarater reliability of measurements.

**Fig. 1:**
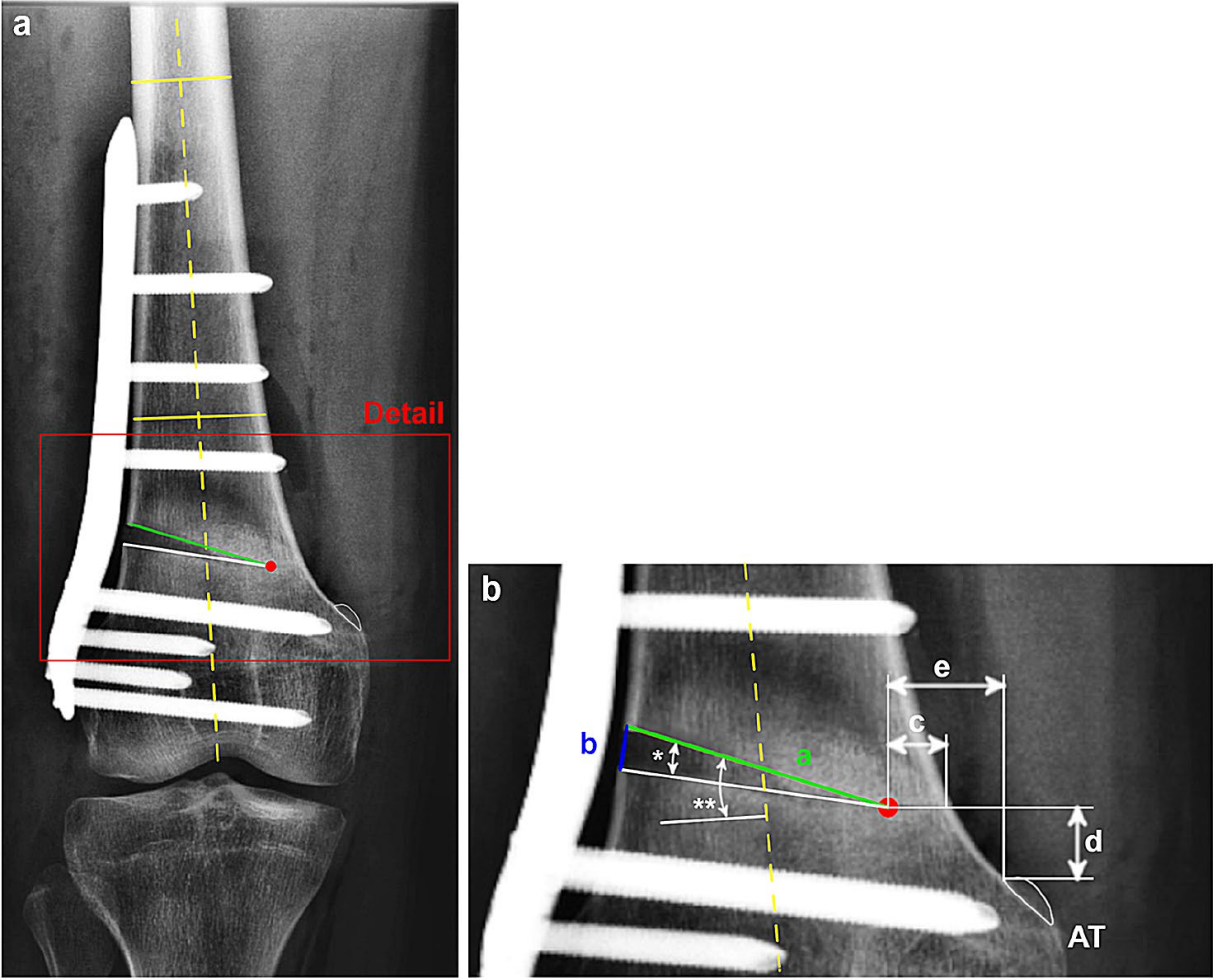
Postoperative radiographic measurements. **a** Standard anterior–posterior radiograph of a right knee after lateral open wedge distal femoral osteotomy using a TomoFix™ (DePuy Synthes, Raynham, MA, USA) locking compression plate. **b** Detailed view of the osteotomy gap with the corresponding measurements. Red dot, osteotomy hinge; *AT* adductor tubercle (encircled in white); Yellow solid lines, medial–lateral connection of the femoral cortical bone; Yellow dashed line, anatomical axis of the femoral diaphysis, defined as the line connecting the midpoints of the two yellow solid lines; Green line (distance “**a**”), length of the osteotomy gap; Blue line (distance “**b**”), height of the osteotomy gap; Distance “**c**”, horizontal distance between the medial cortical bone and the osteotomy hinge; Distance “**d**”, vertical distance between the proximal margin of the AT and the osteotomy hinge; Distance “**e**”, horizontal distance between the proximal lateral margin of the AT and the osteotomy hinge; *(angle alpha), wedge angle between the proximal and distal osteotomy plane; **(angle beta), slope of the osteotomy defined as the angle between a perpendicular line to the anatomical axis of the femur and the proximal osteotomy plane

Measurements were performed as follows: First, the osteotomy hinge was identified and the anatomical axis of the femoral diaphysis was determined. In a detailed analysis, the length (distance “a”) and height (distance “b”) of the osteotomy gap as well as the wedge angle (angle alpha) and the slope of the osteotomy (angle beta) were measured. Additionally, the position of the osteotomy hinge was quantified by measuring the horizontal distance between the hinge and the medial cortical bone (distance “c”) as well as the vertical (distance “d”) and horizontal (distance “e”) distances between the hinge and the proximal margin of the AT. A detailed description of the performed measurements is shown in Fig. 1.

**Fig. 2:**
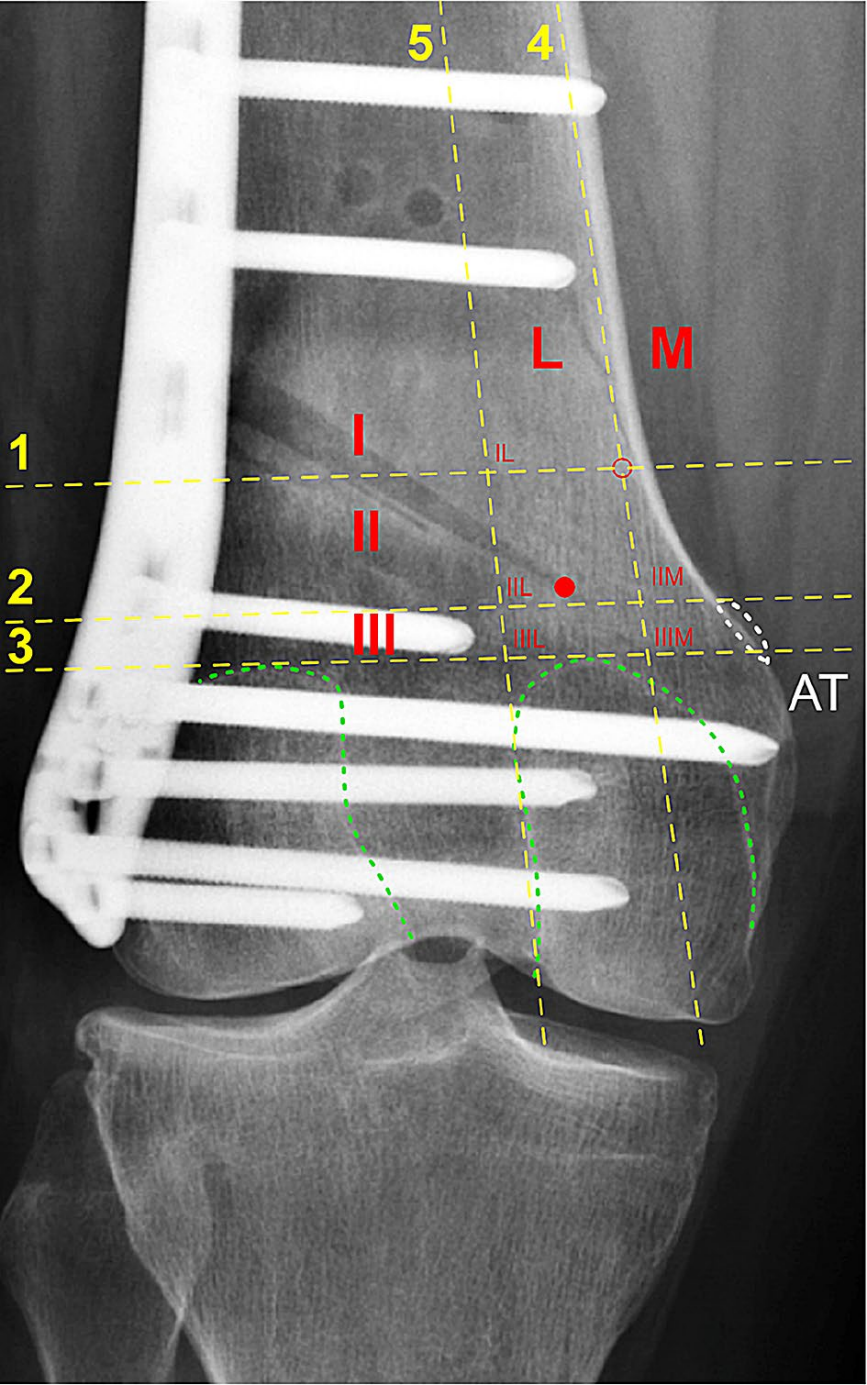
Five-sector grid. *AT*, adductor tubercle (encircled in white); *L*, lateral; *M*, medial; *I*, row 1; II, row 2; III, row 3; Green dashed lines, posterior part of the medial and lateral femoral condyle; Red dot, osteotomy hinge; Red circle, inflection point, defined as the point at which the distance between the medial cortical bone and Line 4 reaches 2 mm; Line 5, tangential to the lateral facet of the medial femoral condyle; Line 4, tangential to the medial femoral cortical bone; Line 3, tangential to the apices of the posterior part of the medial and lateral femoral condyle; Line 2, parallel to Line 3 and crossing the proximal part of the AT; Line 1, parallel to Line 3 and crossing the inflection point

Furthermore, the position of each osteotomy hinge was assigned to a corresponding sector of an established five-sector grid. Distinct bony landmarks (medial femoral cortical bone, femoral condyles, AT) were used to define three rows (I, II, III) and two columns (M, L) of the grid. According to the respective row and column, the sectors were termed IL, IIL, IIIL, IIM, IIIM. Given the definition of the grid, there is no sector IM. A detailed description of the five-sector grid is presented in Fig. 2. The allocation of the hinge position to the corresponding sector was performed by two observers (P.W.W., M.J.F) in agreement with each other.

ICC values of measurements indicated good to excellent intrarater reliability (distance „a “, 0.997; distance “b”, 0.883; distance “c”, 0.966; distance “d”, 0.982; distance “e”, 0.983; angle alpha, 0.786; angle beta, 0.945) and moderate to excellent interrater reliability (distance “a”, 0.788; distance “b”, 0.875; distance “c”, 0.681; distance “d”, 0.952; distance “e”, 0.819; angle alpha, 0.637; angle beta, 0.959).

### Statistical analysis

An a priori power analysis, performed with G*Power (Erdfelder, Faul, Buchner, Lang, HHU Düsseldorf, Düsseldorf, Germany) [8], revealed a total sample size of 90 subjects to detect a difference of 1 mm of the hinge position at an assumed effect size of 0.6 in order to achieve a statistical power of 0.8.

SPSS software version 26.0 (IBM-SPSS, New York, USA) was used for statistical analysis. The level of significance was defined as *p* < 0.05. Categorical variables were reported as count and percentages. Continuous variables were calculated as mean ± standard deviation. Normal distribution of continuous variables was assessed by the Shapiro–Wilk-Test. Group comparison of categorical variables was performed with the Chi-square test or the Fisher’s exact test, as appropriate. Group comparison of continuous variables was performed with the Mann–Whitney *U* test or an unpaired *t* test, as appropriate.

A binary logistic regression was performed to determine the odds of sustaining a medial cortical hinge fracture. The event "medial cortical hinge fracture" (no vs. yes) was defined as the dependent variable. Independent variables which describe the hinge position and demonstrated a significant difference (*p* < 0.05) between the two groups (hinge fracture vs. no hinge fracture) in univariate analysis, were used as the covariates.

## Results

### Incidence and fracture morphology

**Fig. 3:**
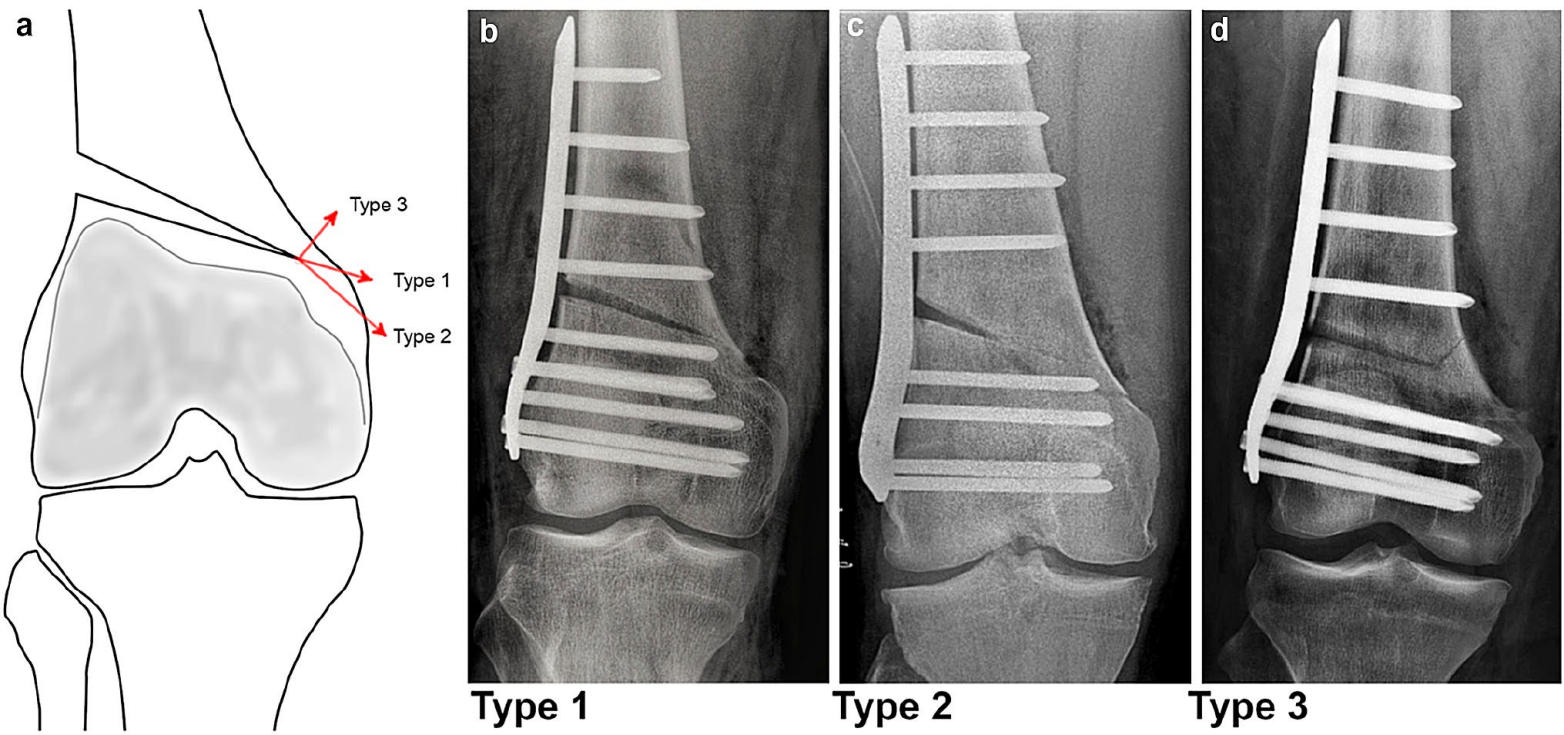
Morphology and classification of medial cortical hinge fractures. **a** Schematic illustration of the three different fracture types. **b** Type 1 fracture, extension of the osteotomy plane. **c** Type 2 fracture, distal to the osteotomy hinge. **d** Type 3 fracture, proximal to the osteotomy hinge

The overall incidence of medial cortical hinge fractures in LOW-DFO was 46%. Three different fracture types could be observed (Fig. 3). The most frequently observed fracture morphology was type 1 (extension, 76%), followed by type 3 (proximal, 20%) and type 2 (distal, 4%).

### Hinge fracture versus no hinge fracture

**Fig. 4:**
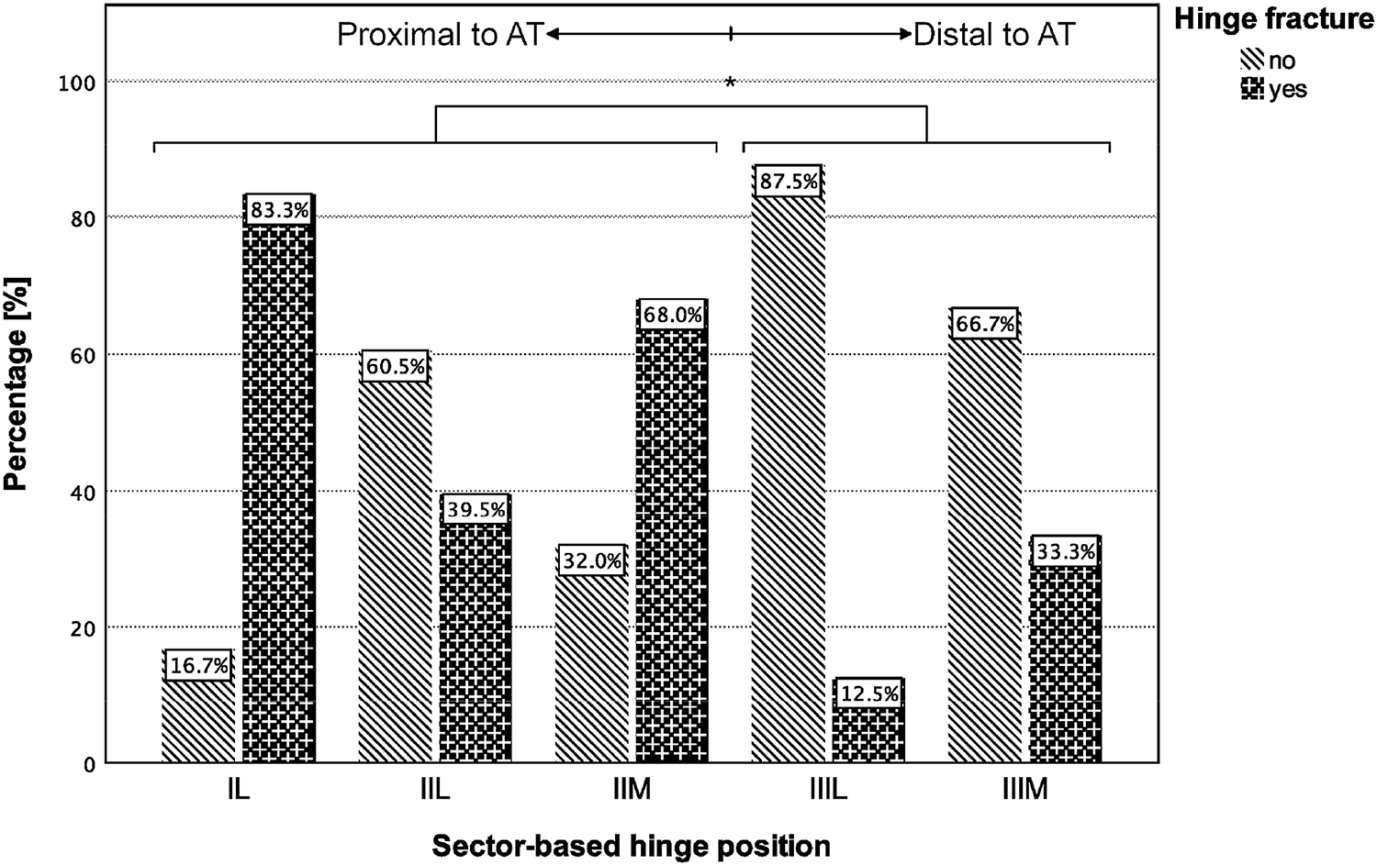
Group comparison of the hinge position (hinge fracture vs. no hinge fracture). Percentages are given for the respective sector; *AT*, adductor tubercle; *statistically significant more hinge fractures for a position of the osteotomy hinge proximal to the AT compared to a position distal to the proximal margin of the AT (level of significance, *p* < 0.05)

A detailed summary of the group comparison is shown in Table 2. For postoperative measurements, a statistically significant difference between the two groups could be observed for the height of the osteotomy gap (distance “b”, *p* = 0.001) and the wedge angle (alpha, *p* = 0.036). Considering the position of the osteotomy hinge, there was a statistically significant difference between the groups for the horizontal distance to the medial cortical bone (distance “c”, *p* = 0.036) and the vertical distance to the proximal margin of the AT (distance “d”, *p* = 0.002). Additionally, a statistically significant difference for the sector-based hinge position (*p* = 0.006; Table 3) and for the hinge position in relation to the proximal margin of the AT (proximal vs. distal; *p* = 0.023) between the hinge fracture group and the no hinge fracture group could be observed (Fig. 4). The incidence of medial cortical hinge fractures was 53% when the osteotomy hinge was located proximal to the AT and 27% when its location was at the level of or distal to the proximal margin of the AT.

**Table 2:** Group comparison (hinge fracture vs. no hinge fracture)

Variable	Hinge fracture		*p* value
	No	Yes	
Number of patients, *n*	54	46	–
Age^a^ (years)	28.7 ± 10.8	34.3 ± 14.2	n.s
BMI (kg/m^2^)	26.7 ± 4.9	25.9 ± 5.0	n.s
Sex			
Male, *n* (%)	19 (35.2%)	21 (45.7%)	n.s
Female, *n* (%)	35 (64.8%)	25 (54.3%)	
Laterality			n.s
Right, *n* (%)	30 (55.6%)	27 (58.7%)	
Left, *n* (%)	24 (44.4%)	19 (41.3%)	
Distance a [mm]	55.8 ± 12.9	53.1 ± 12.8	n.s
Distance b [mm]	5.6 ± 2.2	6.9 ± 1.9	0.001*
Distance c [mm]	8.8 ± 4.2	7.2 ± 5.1	0.036*
Distance d [mm]	4.9 ± 8.5	10.6 ± 9.4	0.002*
Distance e [mm]	14.5 ± 6.4	17.0 ± 7.7	n.s
Alpha [°]	6.1 ± 2.6	7.1 ± 2.6	0.036*
Beta [°]	25.5 ± 5.8	23.6 ± 7.0	n.s
Concomitant procedures^b^			n.s
None, *n* (%)	30 (51.7%)	18 (36.7%)	
HTO-MCW, *n* (%)	2 (3.4%)	2 (4.1%)	
Ligament surgery, *n* (%)	16 (27.6%)	20 (40.8%)	
PF prosthesis, *n* (%)	2 (3.4%)	5 (10.2%)	
Cartilage surgery, *n* (%) Trochleaplasty, *n* (%)	6 (10.3%) 2 (3.4%)	4 (8.2%) 0 (0%)	
Complications			
None, *n* (%)	52 (96.3%)	43 (93.5%)	n.s
Infection, *n* (%)	0 (0%)	2 (4.3%)	
Non-union, *n* (%)	2 (3.7%)	0 (0%)	
Traumatic femur fracture, *n* (%)	0 (0%)	1 (2.2%)	

Categorical variables are presented as count and percentage; Continuous variables are presented as mean ± standard deviation; Positive values of distance “d” indicated a hinge position proximal to the adductor tubercle (AT), while negative values indicated a hinge position distal to the proximal margin of the AT

*BMI*, body-mass-index; *HTO-MCW*, high tibial osteotomy medial closed wedge; *n.s.*, non-significant; *PF*, patellofemoral

^a^Age at surgery

^b^Total number of patients exceeds 100 (total study group), 7 patients had two concomitant procedures

^*^Statistically significant difference between groups (level of significance, *p* < 0.05)

**Table 3:** Group comparison of the sector-based hinge position (IL, IIL, IIIL, IIM, IIIM)

Variable	Sector-based hinge position					*p* value	
	IL	IIL	IIIL	IIM	IIIM		
Number of patients, *n*	6 (6.0%)	43 (43.0%)	8 (8.0%)	25 (25.0%)	18 (18.0%)	–	
Hinge fracture							
Yes, *n* (%)	5 (83.3%)	17 (39.5%)	1 (12.5%)	17 (68.0%)	6 (33.3%)	0.006*	
No, *n* (%)	1 (16.7%)	26 (60.5%)	7 (87.5%)	8 (32.0%)	12 (66.7%)		
Fracture morphology^a^							
Type 1 (extension), *n* (%)	5 (100%)	8 (47.1%)	0 (0.0%)	16 (94.1%)	6 (100%)	n.s	
Type 2 (distal), *n* (%)	0 (0.0%)	1 (5.9%)	0 (0.0%)	1 (5.9%)	0 (0.0%)		
Type 3 (proximal), *n* (%)	0 (0.0%)	8 (47.1%)	1 (100%)	0 (0.0%)	0 (0.0%)		

Categorical variables are presented as count and percentage

*n.s.* non-significant

^a^Hinge fracture group (*n* = 46)

^*^Statistically significant difference between groups (level of significance, *p* < 0.05)

A binary logistic regression model for the occurrence of the event “medial cortical hinge fracture” showed statistical significance for the model itself (*p* = 0.001) as well as for the height of the osteotomy gap (distance “b”; *p* = 0.036), and for the vertical distance to the proximal margin of the AT (distance “d”; *p* = 0.032). An increase of the osteotomy height (distance “b”) and the vertical distance to the AT (distance “d”) by 1 mm, increases the odds of sustaining a medial cortical hinge fracture by 41% and 11%, respectively (Table 4).

**Table 4:** Binary logistic regression model for the event “hinge fracture (no vs. yes)”

Variable	Reference	*p* value	OR	95% CI
Sector-based hinge position				
IL	IIL vs IL	n.s	4.28	0.36–51.10
IIL	Ref	Ref	Ref	Ref
IIIL	IIL vs IIIL	n.s	1.27	0.08–20.10
IIM	IIL vs IIM	n.s	3.10	0.94–10.27
IIIM	IIL vs IIIM	n.s	2.63	0.36–19.18
Distance b	–	0.036*	1.41	1.02–1.94
Distance c	–	n.s	0.97	0.86–1.10
Distance d	–	0.032*	1.11	1.01–1.21
Angle alpha	–	n.s	0.94	0.73–1.21

For the categorical variable, sector-based hinge position, sector IIL was considered as the reference sector, since the osteotomy hinge was localized most frequently in sector IIL. The distances and angles were entered in millimeters and degrees, respectively

*CI*, confidence interval; *n.s.*, non-significant; *OR*, odds ratio; *Ref*, reference

^*^Statistically significant (level of significance, *p* < 0.05)

## Discussion

The present study provides evidence for two major findings; first, with an incidence of 46%, a medial cortical hinge fracture in LOW-DFO is a common finding, which presents itself in three distinct types. In spite of the high incidence, there is no difference in the complication rate for patients with and without a medial cortical hinge fracture. Second, a more proximal position of the osteotomy hinge leads to a higher incidence and increased odds of medial cortical hinge fractures. It is therefore recommended to aim for a position of the osteotomy hinge in sector IIIL or IIIM (at the level of or distal to the proximal margin of the AT) according to the presented five-sector grid when performing a LOW-DFO.

Fractures of the lateral cortical hinge after medial open wedge HTO have been extensively investigated. With an incidence of 18–50% [4, 12, 16, 18, 19, 22–25, 28, 33], lateral cortical hinge fractures in HTO are a common finding and have been associated with the occurrence of non-unions of the osteotomy gap [4, 12, 23]. Similar results have been shown for DFO. The incidence of lateral/medial cortical hinge fractures is reported to be almost 48% and 39% for the MCW [10] and the LOW [20] technique, respectively. This is in accordance with an overall incidence of 46% of medial cortical hinge fractures in LOW-DFOs in the present study. Non-unions of the osteotomy gap are dreaded complications of LOW-DFO [2, 3, 6, 14, 20, 30] and may be attributed to medial cortical hinge fractures and the associated reduced axial and torsional stiffness as well as the increased rotational movement across the osteotomy gap for the bone-implant construct [1, 29]. However, despite the high incidence of hinge fractures in the present study, the complication rate was only 5% and showed no difference between patients with and without a medial cortical hinge fracture.

In 2012, Takeuchi et al. [33] proposed a classification for lateral cortical hinge fractures in medial open wedge HTO. In a retrospective analysis of 104 medial open wedge HTOs, 26 (25%) fractures of the lateral cortical hinge with three different fracture types were observed [33]. Similarly, three distinct types of medial cortical hinge fractures have been observed in LOW-DFO in the present study (Fig. 3). With an incidence of 76% among the hinge fractures, fractures in extension of the osteotomy gap (type 1) were most common. According to the distribution of fracture types for the present study, fractures in extension of the osteotomy gap are also most frequently observed in medial open wedge HTOs [12, 18, 24, 25, 28, 33].

Since distinct fracture types (Takeuchi type 2 and type 3) after medial open wedge HTOs are associated with an increased complication rate [12, 25, 33], many efforts have been made to establish a safe zone for the position of the osteotomy hinge in order to reduce the risk of hinge fractures [13, 24, 28]. A recently published cadaveric analysis of lateral cortical hinge fractures in MCW-DFO concluded that the ideal position of the osteotomy hinge is at the upper border of the lateral femoral condyle to minimize the risk of unstable lateral cortical hinge fractures [15]. This conclusion was supported by two main findings. First, the upper border of the lateral femoral condyle is located within the femoral attachment site of the lateral gastrocnemius head. As a result, the femoral attachment of lateral gastrocnemius head acts as a soft tissue stabilizer of the osteotomy hinge [15]. Second, an area of low bone density was observed in the region of the upper border of the lateral femoral condyle. The authors assumed that this area tolerates increased plastic deformation and thus reduces the risk of lateral cortical hinge fractures [15]. A second biomechanical study showed similar results with a higher incidence and increased propensity for instability of lateral cortical hinge fractures in MCW-DFO with a supracondylar hinge position compared to a condylar hinge position [26]. Consistent to the proposed safe zones for the MCW technique, a condylar hinge position at the level of or distal to the proximal margin of the AT has been shown to reduce the odds of hinge fractures for the LOW technique, as demonstrated in the present study. Whether or not additional soft tissue stabilizers and certain bone density patterns at the distal medial femur contribute to the formation of medial cortical hinge fractures has not been investigated yet and therefore remains a subject of future research.

A biplanar technique for LOW-DFO is recommended to achieve a position of the osteotomy hinge at the level of or distal to the proximal margin of the AT without harming the trochlea groove [9]. Besides the ability of a substantially more distal level of the axial osteotomy, the biplanar technique has several more advantages. One study showed an increased torsional stiffness and reduced rotational movement across the osteotomy gap for the biplanar technique compared to the uniplanar technique, especially when a disruption of the medial cortical bone was simulated [29]. Another important benefit of the biplanar technique is the increased bone-surface area, which is believed to improve bone healing [35]. Osseous consolidation is further supported by the improved healing potential of the metaphyseal compared to the diaphyseal bone [31].

There are some limitations of the present study. Given the absence of clinical outcomes, it is not possible to make a conclusion on whether a fracture of the medial cortical hinge affects the functional and clinical outcomes. Since this study was conducted to analyze the incidence and morphology of medial cortical hinge fractures as well as to describe a safe zone for the osteotomy hinge, collecting and reporting clinical data would have been beyond the scope of this study. Furthermore, the incidence of medial cortical hinge fractures in LOW-DFO was assessed by simple postoperative AP radiographs. However, previous studies investigating lateral cortical hinge fractures in medial open wedge HTO showed that the incidence of hinge fractures is even higher when assessed by computed tomography (CT) [18, 19]. To avoid unnecessary radiation, postoperative CT scans are not routinely acquired, which is why simple radiographs were used for the present study. The proposed five-sector grid may be affected by a malrotation of the knee during image acquisition, which can falsify a reliable assignment of the osteotomy hinge to the corresponding sector. It is important to keep this in mind, especially when the five-sector grid is applied during surgery.

## Conclusion

Medial cortical hinge fractures in LOW-DFO are a common finding with three distinct fracture types. The most frequently observed fracture type was an extension of the osteotomy plane (type 1, 76%). To minimize the risk of medial cortical hinge fractures, it is recommended to aim for a position of the osteotomy hinge at the level of or distal to the proximal margin of the adductor tubercle.
